# A Rare Case of Drug-Induced Sweet Syndrome After Pembrolizumab Therapy

**DOI:** 10.7759/cureus.62027

**Published:** 2024-06-09

**Authors:** Suchitra Muralidharan, Andrew T Mariano, Dhruv Joshi, Trisha E Andrews, Seban Liu, Ahsan Basha, Nha Huynh

**Affiliations:** 1 Internal Medicine, Riverside Medical Center, Kankakee, USA; 2 Cardiology, Riverside Medical Center, Kankakee, USA; 3 Oncology, Riverside Medical Center, Kankakee, USA

**Keywords:** non small cell lung cancer, lung cancer, pembrolizumab cutaneous side effect, pembrolizumab, drug-induced sweet syndrome

## Abstract

Sweet syndrome is an uncommon inflammatory disorder characterized by the abrupt appearance of painful, erythematous papules, plaques, or nodules on the skin. Fever and leukocytosis frequently accompany the cutaneous lesions. In addition, involvement of the eyes, musculoskeletal system, and internal organs may occur. Sweet syndrome has been associated with a broad range of disorders. There are three subtypes: classical Sweet syndrome, malignancy-associated Sweet syndrome, and drug-induced Sweet syndrome. Classical Sweet syndrome is not associated with malignancy or drugs. It is essentially associated with an upper respiratory infection, gastrointestinal infection, inflammatory bowel disease, and pregnancy. Malignancy-associated Sweet syndrome is associated with hematologic malignancy more than solid malignancy, most commonly with acute myeloid leukemia. Drug-induced Sweet syndrome usually develops approximately two weeks after drug exposure, in patients who lack a prior history of exposure to the inciting drug. Here we are discussing our patient, a 68-year-old male who presented eight weeks after starting chemotherapy with pemetrexed, carboplatin, and pembrolizumab for left lung adenocarcinoma with macular rash. On further investigation with biopsy was found to have neutrophilic dermatitis, hence being diagnosed with drug-induced Sweet syndrome. Histopathology revealed a dermis with infiltration of neutrophils with lekocytoclasia.

## Introduction

Drug-induced Sweet syndrome is associated with various drug classes including Antibiotics, Antiseizure medications, Anti-HIV drugs, Antihypertensives, Antineoplastics, Antipsychotics, Antithyroid hormone synthesis drugs, Colony-stimulating factors, Contraceptives, Diuretics, Immunosuppressants, Nonsteroidal anti-inflammatory drugs (NSAIDs), and Retinoids [1-4]. Antineoplastics known to be associated include bortezomib, imatinib mesylate, ipilimumab, lenalidomide, topotecan, and vemurafenib [4]. Checkpoint inhibitor immunotherapy, like pembrolizumab, can trigger neutrophilic dermatoses, more frequently in patients already receiving chemotherapy. This study was presented as a poster presented at the 2023 Illinois Chapter of the American College of Cardiology Fellow-In-Training Poster Presentation on 28th April 2023.

## Case presentation

**Figure 1:**
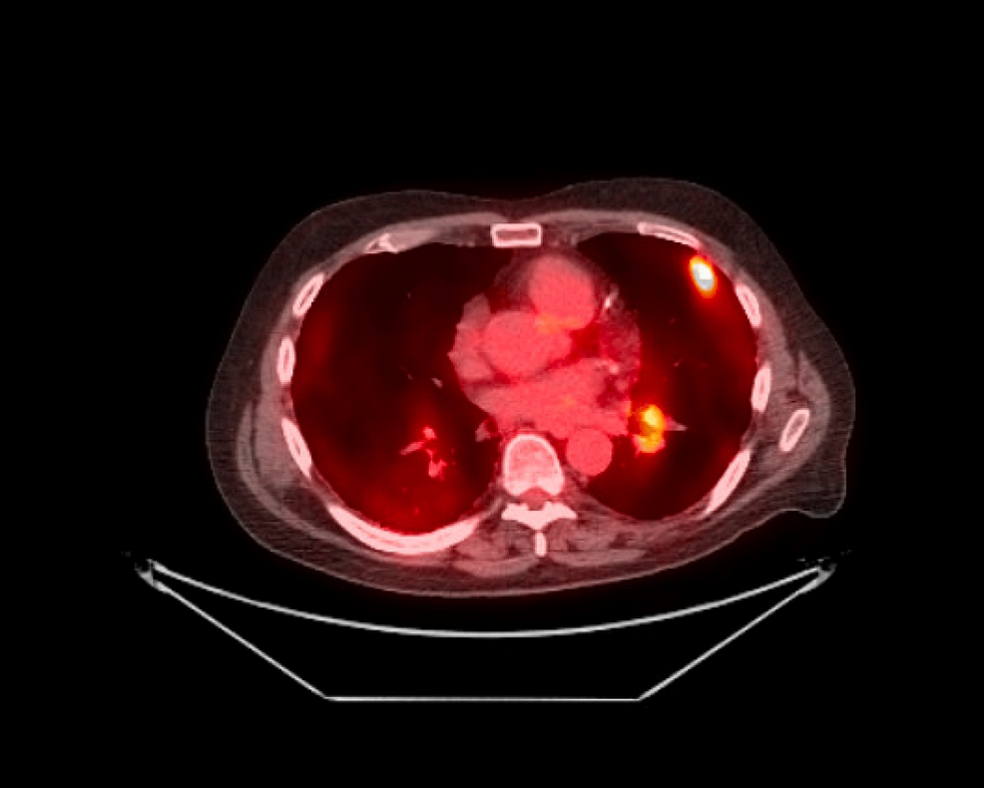
Initial PET-CT scan The positron emission tomography-computed tomography (PET-CT) scan image is showing bright areas corresponding to hyper-metabolic activity in left hilar lymph node and left lower lung base which appeared to be the primary location of lung carcinoma.

A 68-year-old male with 40 plus pack years history of smoking, following lung cancer screening, was identified with multiple spiculated pleural-based nodular areas, advanced parenchymal lung disease with honeycombing at the lung bases, volume loss and retraction with traction bronchiectasis consistent with pulmonary fibrosis, no bulky mediastinal lymphadenopathy. Positron emission tomography-computed tomography (PET-CT) (Figure 1) confirmed hypermetabolic uptake in multiple peripheral pulmonary nodules and left hilar lymph node, and with low metabolic activity in ground glass opacities.

**Figure 2:**
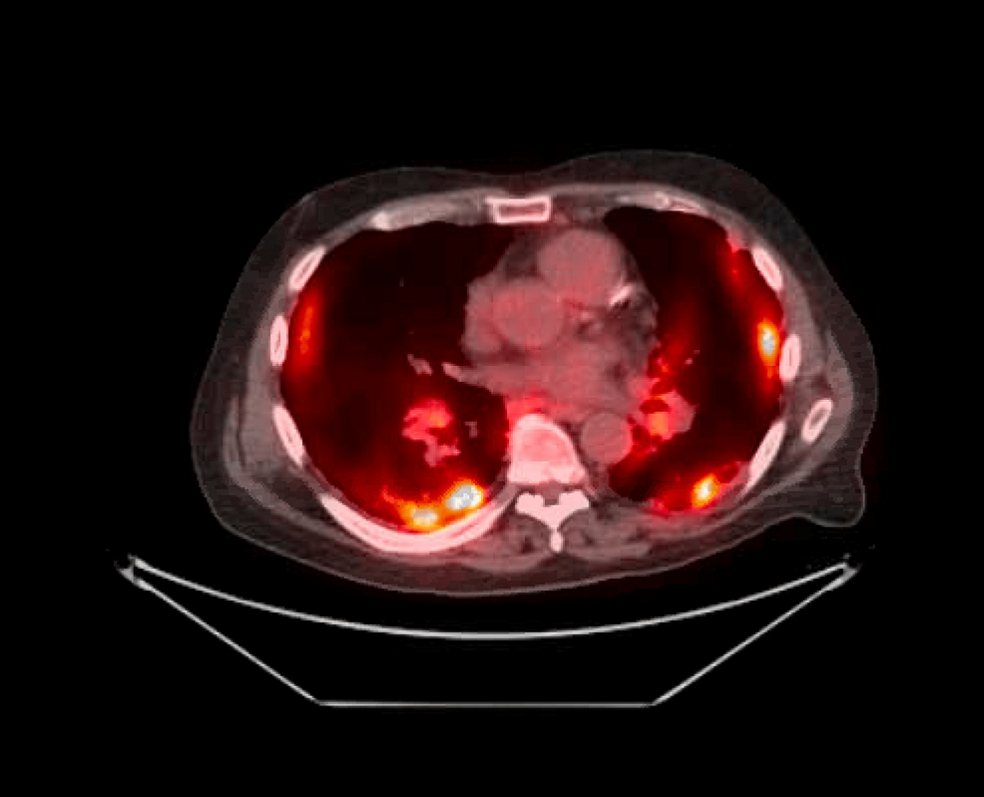
Repeat PET-CT scan The image is showing a mixed response to chemotherapy with hyper-metabolic activity bilaterally in all areas with reduced activity in the primary left lower lung base. PET-CT: Positron emission tomography-computed tomography

CT needle biopsy of the left lower lung indicated adenocarcinoma equivocal for Her-2/Neu-2+ and negative by FISH, PD-L1 TPS 2%, 1+ intensity. The patient completed three cycles of chemotherapy with carboplatin, pemetrexed, and pembrolizumab (WM1), restaging PET-CT (Figure 2) performed on 1/6/2023 showed mixed response with a reduction in size of primary left lower lung mass.

**Figure 3:**
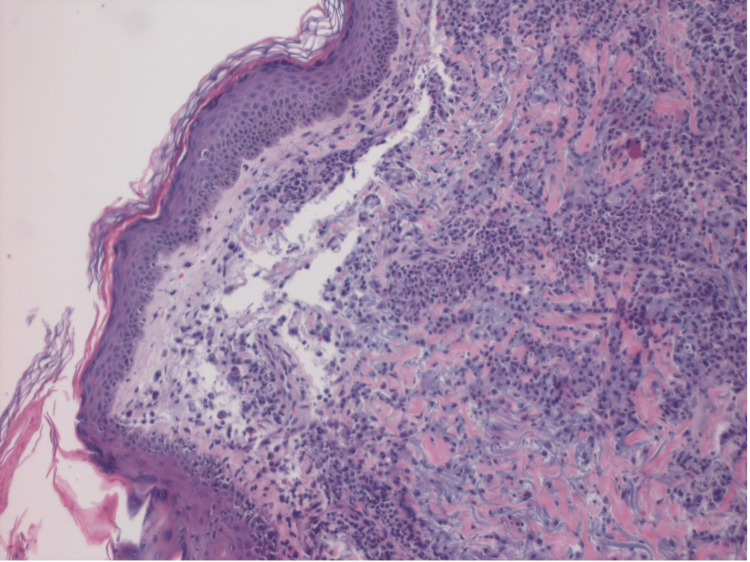
Histopathology image (I) Tissue biopsy from a macular lesion on skin showing infiltration of neutrophils in the dermis.

**Figure 4:**
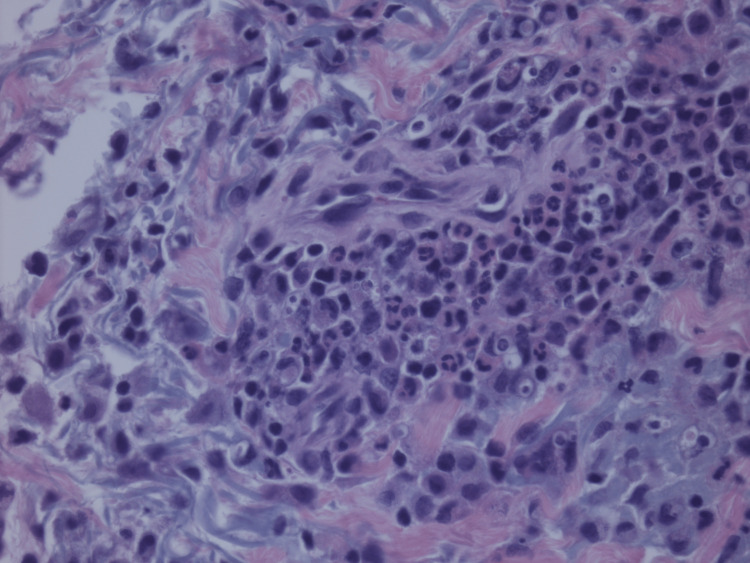
Histopathology image (II) Showing dense infiltration of inflammatory (blue stained) cells in affected tissue in close-up.

The patient presented to the hospital with complaints of shortness of breath, fevers, and chills. In the emergency room, the patient was noticed to be in atrial fibrillation with rapid ventricular rate (RVR). The patient was given a dose of diltiazem and converted spontaneously. Upon admission, the patient was treated for community-acquired pneumonia with ceftriaxone, and azithromycin while also noting acute hypoxic respiratory failure due to pneumonia and ongoing left lung adenocarcinoma. Cardiology was consulted for atrial fibrillation with RVR, and the patient was started on anticoagulation. The patient underwent transthoracic echocardiogram (TTE), which revealed grossly normal left ventricular function with normal diastology. With sputum culture growing gram-positive cocci, was started on vancomycin as well. The patient was eventually started on metoprolol tartrate for rate control and apixaban for anticoagulation. The patient’s antibiotics were then modified to cefepime and doxycycline noting negative *Streptococcus*, Legionella, and methicillin-resistant *Staphylococcus aureus* screening test. On the third day of admission, the patient was noted to have a raised macular rash on the arms, face, and neck. Respiratory pathogen panel, mycoplasma, autoimmune workup, and fungal workup were done and found to be negative. The patient had a few episodes of atrial fibrillation with RVR, and digoxin was added for better rate control. Oncology was consulted, who suggested that if the biopsy of the macular rash suggests an immune-mediated etiology, could consider this a toxicity of pembrolizumab. Antinuclear antibody (ANA) and anti-neutrophil cytoplasmic antibodies (ANCA) panels were ordered and were reported as negative, along with other infectious workups, following which the patient was started on a six-week course of tapering prednisone for presumed pembrolizumab pneumonitis, after collecting samples for hypersensitivity pneumonitis (HP) panel, respiratory immunoglobulin E (IgE) panel, and myositis panel. As the patient’s hospital stay progressed, his oxygen requirements were noted to have increased. The patient was started on dronedarone for better rate management by discontinuing digoxin and metoprolol tartrate was continued. The patient was weaned down to 3L of supplemental oxygen via nasal cannula and was discharged fully understanding that he would have to be compliant with steroids and antibiotics and follow up with oncology, and pulmonology as an outpatient. Pulmonary embolism was ruled out during the hospital as a cause for worsening oxygen requirements. On following an appointment with the oncologist, the patient was advised to start on growth factor support with each cycle of treatment for agranulocytosis secondary to cancer chemotherapy. Two days after discharge from the hospital, at the oncology appointment, the patient’s hyperpigmented areas on the arms were found to be resolving. Ten days after discharge from the hospital, the patient’s hyperpigmentation in his arms was noted to have been resolved and he was started on cycle 1 of docetaxel for progressive lung adenocarcinoma, noting the patient was asymptomatic from pneumonia and having completed most of his antibiotic course, continuing steroid taper. The patient’s pembrolizumab was stopped and changed to docetaxel attributing to atrial fibrillation with rapid ventricular response. This coincided with the resolution of macular rash as well, favoring pembrolizumab as a possible cause for neutrophilic dermatitis noted on the biopsy (Figures 3, 4), as all the work up for autoimmune, fungal causes, hepatitis, pneumocystis, and allergy were resulted as negative.

## Discussion

Pembrolizumab is an antineoplastic agent and an anti-PD1 monoclonal antibody, which is an immune checkpoint inhibitor. It is prescribed for multiple cancers, including non-small cell lung cancer, which our patient had until disease recurrence or unacceptable toxicity. It is administered via infusion. It is especially preferred in patients with non-small cell lung carcinoma with tumors with PD-L1 expression with TPS>/= 1%, which our patient had. The drug is known to cause adverse reactions like immune-mediated pneumonitis which our patient was treated for, uveitis, neurologic toxicity like cerebral hemorrhage, confusion, myasthenia gravis, reversible posterior leukoencephalopathy syndrome, immune-mediated nephritis, autoimmune hemolytic anemia, immune thrombocytopenia, acquired hemophilia, and disseminated intravascular coagulation, immune-mediated colitis, hypothyroidism, hyperthyroidism, adrenocortical insufficiency, myocarditis, pericarditis, and vasculitis. Apart from these, pembrolizumab is also known to cause dermatologic toxicities including Stevens-Johnson syndrome (SJS), toxic epidermal necrolysis (TEN), and bullous pemphigoid [5]. It is known to cause pruritic skin rash with vitiligo. SJS and TEN appear as target-like lesions which then would become confluent, brightly erythematous, and bullous. Bullous pemphigoids appear as erythematous, urticarial, non-inflammatory blisters numerous in numbers. These are distributed in the trunk, extremities flexures, and axillary and inguinal folds. Our patient presented with raised macular rash, non-pruritic, erythematous, non-blister, no vitiligo noted, with irregular borders. Sweet syndrome primarily consists of four features: cutaneous erythematous eruption with papules (raised macules)/plaques, dermal non-vasculitic neutrophilic infiltration on biopsy, fever, and peripheral neutrophilia [6]. It can rarely be associated with cellulitis like subcutaneous and necrotizing fasciitis-like presentation. The cutaneous eruption consists of erythematous to violaceous tender papules which enlarge to form plaques with irregular pseudo-vesicular surfaces. They mostly appear in the upper extremities, face, and neck. In the upper extremities, it appears especially on the dorsum of the hands. Other systemic manifestations can occur depending on the organ involved, including the lungs. It is commonly seen in age group between 30-60 years of age, it can also be seen in infants, children, and older adults in classic Sweet syndrome. The age of onset can be older in malignancy-associated Sweet syndrome. In Sweet syndrome associated with drugs the percentage of patients who are women is estimated to be 70%. There is no obvious racial predilection. There are diagnostic criteria that help in the diagnosis of drug-induced Sweet syndrome [7]: A. Abrupt onset of painful erythematous plaques or nodules, B. Histopathologic evidence of a dense neutrophilic infiltrate without evidence of leukocytoclastic vasculitis, C. Pyrexia >38°C, D. Temporal relationship between drug ingestion and clinical presentation, or temporally-related recurrence after oral challenge, E. Temporally-related resolution of lesions after drug withdrawal or treatment with systemic corticosteroids.

All five criteria have to be satisfied to be considered as drug-induced Sweet syndrome. Based on this it can be suggested that pembrolizumab could have caused Sweet syndrome in the patient discussed above. Treatment for toxicities from immune checkpoint inhibitor therapy is based on grades of toxicities [8]. Grade 1 toxicity is essentially without symptoms, where therapy can be continued with immune checkpoint therapy. Grade 2 toxicity would affect the quality of life, requiring intervention, where corticosteroids may be administered. Grade 3 toxicity would be a failure to respond to indicated intervention for Grade 2 dermatitis; treatment would be holding immune checkpoint therapy, consulting dermatologists, starting systemic corticosteroids, and considering alternative immune checkpoint therapy after consulting oncology. Grade 4 toxicity would be rashes unmanageable with prior intervention and would be managed by permanent discontinuation of immune checkpoint therapy [9]. The patient recovered after the discontinuation of pembrolizumab and after placing him on oral steroid taper and was started on docetaxel, an antineoplastic agent since he had other complications as well [8,9].

## Conclusions

Pembrolizumab-associated cutaneous erythematous eruption form of Sweet syndrome is rare and has not been reported before. Tissue biopsy is a good diagnostic test, once all other causes of neutrophilic dermatitis have been ruled out. Early therapy with glucocorticoids after excluding other possible causes would be beneficial in resolving this condition.
